# Occupational noise exposure at sea: A socio-legal study on fish harvesters’ perceptions in Newfoundland and Labrador, Canada

**DOI:** 10.3389/fpubh.2023.1092350

**Published:** 2023-04-17

**Authors:** Om Prakash Yadav, Desai Shan, Atanu Sarkar, Lorenzo Moro

**Affiliations:** ^1^Division of Community Health and Humanities, Memorial University of Newfoundland, St. John’s, NL, Canada; ^2^Faculty of Engineering and Applied Science, Memorial University of Newfoundland, St. John’s, NL, Canada; ^3^SafetyNet Centre for Occupational Health and Safety Research, Memorial University of Newfoundland, St. John’s, NL, Canada

**Keywords:** noise exposure, hearing loss, occupational health and safety, fish harvesters, noise prevention, policies and regulations

## Abstract

**Introduction:**

Noise is a significant health hazard for fish harvesters. Chronic exposure to hazardous noise levels of 85 dB (A) for an 8-h work shift can have adverse health impacts, including both auditory and non-auditory health problems such as noise-induced hearing loss, stress, hypertension, sleeping disorders, and impaired cognitive performance.

**Methods:**

A review of legislation and policies governing workplace noise exposure, as well as qualitative, semi-structured interviews, were conducted to assess how fish harvesters in Newfoundland and Labrador (NL) manage onboard occupational noise exposure and perceive noise-induced health problems, as well as the barriers and challenges associated with preventing and controlling noise exposure.

**Results:**

The legal review shows no compulsory noise preventive measure at the fishing vessel design stage in Canada. Limited implementation of *Occupational Health and Safety (OHS) regulations* to control and prevent onboard noise by employers in Newfoundland and Labrador. Fishers reported that their workplace is noisy. Over time, fish harvesters adapted to the environment and learned to tolerate loud noise, displaying fatalistic behavior. Fish harvesters reported avoiding using hearing protection onboard due to navigation safety concerns. Fishers reported hearing loss as well as other non-auditory health problems. Inadequate noise control measures adopted by employers, a limited supply of hearing protection onboard, and a lack of regular hearing testing, training, and education were identified as the main barriers to preventing and controlling noise exposure.

**Conclusion:**

Proper implementation of NL *OHS regulations* and the development of hearing conservation initiatives by employers are necessary. All stakeholders, including the federal and provincial governments, WorkplaceNL, and not-for-profit fishing organizations in the province, are strongly recommended to initiate training and education campaigns to help fish harvesters understand noise exposure and adopt preventive measures.

## 1. Introduction

Fishing is a prominent sector in many countries and has had a considerable impact on the expansion and development of the blue economy, which is the sustainable utilization of marine resources for growth in the economy, enhanced livelihood, and employment generation while conserving the health of ocean ecosystems (1, 2). Commercial fish harvesters face various risk factors related to health and safety. These risk factors include but are not limited to various physical and mental health concerns, such as musculoskeletal disorders, hearing loss, psychological distress, and sleep disturbances (3–5). Occupational noise exposure is recognized as a serious risk factor for the health and well-being of fish harvesters (4, 6–8). Long-term exposure to harmful noise levels is a documented contributing factor to tinnitus and Noise-Induced Hearing Loss (NIHL) (4, 6, 8–11). In addition to auditory health impacts, chronic noise exposure also leads to many non-auditory conditions, such as sleep disturbances, fatigue, anxiety, stress, and cardiovascular and gastric disorders (5, 8, 12). Statistics from the WorkplaceNL, a provincial governmental agency in Newfoundland and Labrador that administers workplace health, safety, and workers’ compensation and offers services to employers, injured employees, and dependents, show that fish harvesters were among the top groups of workers who filed hearing loss claims (13). According to recent research conducted among NL fish harvesters, the majority of participants are exposed to loud noises during various fishing activities. This research revealed that noise exposure (L_EX,8h_) on eight of the 12 fish vessels was detected more than 85 dB(A) (14).

In Canada, provincial governments are in responsible of protecting workers’ occupational health and safety. The occupational health and safety (OHS) act and its regulations cover occupational noise exposure management methods at the workplace. The *Occupational Health and Safety Regulations, 2012*, under the *Occupational Health and Safety Act*, 1992 covers health and safety issues in NL. These regulatory standards adopt the American Conference of Industrial Hygienists’ criteria. The maximum allowable noise at a workplace is set as 85 dB(A) for 8 h of a work shift (15, 16). The fundamental problem with this regulation is that it is based on an 8-h workday and presumes that the worker spends the rest of their time at home, where noise levels are generally low and are not controlled by this Act because it is not a workplace. But what if the worker spends most of their after-work time in a cabin or kitchen with noise levels of 75 dB (A) or 80 dB (A)? The worker is still on the job, and the noise levels are not beyond the maximum noise limits, but they are much over the International Maritime Organization [IMO] standards, where the maximum noise levels in the crew accommodation should be 60–75 dB (A) [1,600 up to 10,000 gross tonnage (GT)] and 55–75 dB (A) [≥10,000 GT] (17). In accordance with the regulations, the employer shall take necessary steps to implement control measures in place to limit noise to recommended levels; if it is not practical to do so then need to isolate employees from the noise, or the workers should wear personal protection equipment (PPE) (16).

The workers’ awareness of the negative impacts of exposure to loud noise is another aspect of workplace noise governance. According to a survey conducted among NL fish harvesters, the skippers of small fishing boats were unaware of the risks presented by noise sources onboard (14).

Various federal and provincial organizations in Canada oversee and regulate fisheries, such as Fisheries and Oceans Canada (DFO), Transport Canada (TC), and local governments. According to the DFO’s statistics, small fishing vessels make up around 99% of all fishing vessels in use in NL (18). However, there are no federal or provincial regulations in Canada that mandate a maximum noise level onboard small fishing boats that are less than 24.4 meters in length overall (LOA) and not more than 150 GT (14). According to the IMO’s adoption of the code on noise levels on board ships, larger boats have explicit noise exposure limitations for all locations onboard, however small vessels are frequently overlooked and noise levels are usually monitored against the 85 dB standard (A) (17). Structural design is also key stage to eliminate and control noise level on board fishing vessels (17). Federal regulations covers vessel safety and design in Canada. However, fishing vessels safety regulations in Canada do not cover the management of onboard noise exposure at the operational stage (19, 20).

The economy of NL is heavily reliant on the fishery sector (21). According to WorkplaceNL data on fishing safety, six fatalities were recorded in 2020, and the incidence of lost-time injuries per 100 workers was higher than the provincial average of 1.6. Fish harvesters had a 13-fold higher risk of death on the work, a 4-fold higher risk of suffering a severe injury, and a 2-fold higher risk of losing their hearing.48 Nl Fish harvesters filed 8.3% of hearing loss cases between 2011 and 2017 (13).

In Newfoundland and Labrador, occupational health and safety regulations apply to all employers, with no difference made between land-based and maritime workers. Fish harvesters labor in a confined and moving environment, and they are subject to constant noise exposure while working and resting during multi-day fishing trips. As a consequence, fish harvesters are more vulnerable to noise exposure and associated risks; however, no specific legislation addresses noise exposure levels and safety precautions aboard fish vessels. To address this issue, a legal regulatory review was conducted to examine the current governance of fisheries in Canada to manage onboard noise exposure and observe the potential gaps in the existing regulations. A qualitative study was also conducted to explore how NL fish harvesters manage onboard noise exposure, limit noise-induced health problems, and identify potential barriers and obstacles to preventing onboard noise exposure.

## 2. Materials and methods

### 2.1. Legal regulatory review

A legal regulatory review was performed to investigate the onboard noise exposure regulations applicable to fishing vessels in Canada. This paper includes legal doctrinal analysis and legal sources to provide a precise scientific representation of the relevant regulatory regimes. The regulatory review explores fishing governance, occupational noise exposure, and related regulations in NL and Canada. The study also analyzes the potential gaps in the current health and safety regulations in NL. The following organization’s official websites were explored to find the relevant regulations: Fisheries and Oceans Canada; Transport Canada; Government of Newfoundland and Labrador; Department of Fisheries and Aquaculture; WorkplaceNL; Professional Fish Harvesters Certification Board; NL-Fish Harvesting Safety Association; Fish Food Allied Workers-Union Unifor; and Fisheries and Marine Institute of Memorial University of Newfoundland.

### 2.2. Qualitative study

A qualitative research was conducted to explore the noise risk perception among NL fish harvesters. At the beginning of the study, 30 interviews were planned but due to the current COVID-19 situation, only 12 telephonic interviews were conducted. NL fish harvesters who were 18 years or older, held a license issued by the Professional Fish Harvesters Certification Board (PFHCB), and had one or more years of fishing experience were included in the interviews. Fish harvesters who had previously worked in a noisy environment other than the fishing industry for one year or more and who had been diagnosed with a hearing issue were excluded from the study. The fish harvesters interviewed had experience ranging from 3 to 60 years and worked in various positions such as deckhands, second mates, skippers, owners, and operators on small to large fish vessels ranging from 12.5 feet to 160 feet. Most fish harvesters are involved in crab, lobster, cod, and capelin fishing.

A semi-structured interview guide was prepared to collect the data. Questions about hearing loss and other general health problems caused by noise and difficulties in preventing noise exposure and noise-related health problems were addressed. Ethical approval was obtained from the Interdisciplinary Committee on Ethics in Human Research, Memorial University of Newfoundland (file number: 20210888) in November 2020. A recruitment flyer with eligibility criteria and other research material was distributed across various social media platforms. Additionally, local fishing organizations, including the PFHCB, Newfoundland and Labrador-Fish Harvesting Safety Association (NL-FHSA), and Fish, Food and Allied Workers Union-Unifor (FFAW-Unifor) were contacted to disseminate the research information on their websites. Qualitative data was collected through telephonic interviews. The duration of the interviews ranged from 1 to 2 h. A pilot study with two participants was done to evaluate the feasibility and suitability of the interview questionnaire before executing the main research. Data collection was conducted between January to April 2021. All the interviews were audio-recorded for transcription and future data analysis. Thematic analysis method was used for the data analysis. The health capital method was adopted to comprehend how fishers perceive noise exposure and explore potential barriers to alleviating noise-related health issues.

## 3. Results

### 3.1. Governance of noise on fishing vessels

Transport Canada (22), Fisheries and Oceans Canada (23), the Canadian Coast Guard (24), and the Transportation Safety Board of Canada (TSB) (25) are the primary government agencies responsible for fishing governance, including issuing licenses, certifying fishing vessel navigation personnel, registering of fishing vessels, providing safety training and navigational aids, investigating accidents involved fishing vessels and regulating fish harvesting quotas. They also manage security, environmental protection, pollution control, and marine investigations. TC implements and manages fishing vessel safety regulations in Canada. *Fishing Vessel Safety Regulations* (19) [applicable for fish vessels, not more than 24.4 meters and not more than 150 GT] and *Large Fishing Vessel Inspection Regulations* (20) [applicable for fish vessels over 24.4 meters and over 150 GT] are currently in force in Canada. These regulations cover safety measures required in fish vessel design and structure. Noise exposure is a significant risk factor directly or indirectly associated with ship design and construction; however, the fishing vessel safety vessel regulations do not cover any noise mitigation measures that can minimize onboard noise exposure.

Fishing organizations in Newfoundland and Labrador, such as the Department of Fisheries, Forestry and Agriculture (26), WorkplaceNL (27), Professional Fish Harvesters Certification Board (28), NL-Fish Harvesting Safety Association (29), Fish Food Allied Workers-Union Unifor (30), and Fisheries and Marine Institute of Memorial University of Newfoundland (31) take care of various issues related to fisheries in the region. The Department of Fisheries, Forestry, and Agriculture is focused on the growth and advancement of the fishing industry. The Department collaborates with several partners to ensure the long-term development of fishing sectors in the NL (26). WorkplaceNL (27) provides insurance coverage, including hearing loss claims and a no-fault environment to workers and management across the province. The PFHCB supports fish harvesters by advancing the profession’s interests, managing fish harvester’s registry, creating, assessing, and recommending professionalization courses, identifying requirements, and issuing licenses to eligible fish harvesters (28). The NL-FHSA supports fish harvesters by promoting and disseminating best practices within the fishing sector, supporting an effective stakeholder counseling system, and facilitating a joint security approach through safety education and information exchange (29). FFAW supports fishermen by addressing shipping concerns, keeping the crew up to date on price and limit details, and promoting the fleet in negotiations (30). The Marine Institute offers a range of training programs to fish harvesters through several community-based education initiatives supported by the provincial and federal government (31).

NL OHS regulations cover the noise hazard regulations and describes the requirements which need to be followed by the employers (16). According to the NL OHS Regulation, if the noise level in a workplace exceeds the acceptable threshold, the employer must take reasonable measures to control the noise and provide protective equipment if noise cannot be reduced. NL OHS Regulation follows the American Conference of Governmental Industrial Hygienists’ Noise Threshold Limit Values, which are 85 dB(A) with a doubling rate of 3 dB(A) (32). For example, 85 dB(A) is applicable for 8 h. If the noise level rises to 88 dB(A), the work period should be reduced to 4 hours. Employers should develop and maintain hearing conservation programs that include a survey to identify high noise zones, yearly hearing tests for all employees, hearing tests every 3 months for new employees, and mandatory training and education for all employees to identify the health effects of noise and proper use of hearing protectors (16). The requirements apply to all workers, regardless of the nature of their work environment. There are no special criteria or guidelines for the fish harvesters who operate fish vessels in the province.

### 3.2. Health capital: A theoretical framework to understand fishing OHS challenges

The Health Capital approach was adopted to analyze the collected data. Health capital includes field-specific skills, competencies, social connections, financial resources, and prestige that may be used directly or through conversion from other types of capital to maintain good health and control illness. It, therefore, draws on and enhances the synergy of economic, social, cultural, and symbolic capital (33). Health capital is one approach to evaluating risk in an individual’s characteristics and recognizing gaps such as a lack of skill, education, or experience for qualitative risk assessment. Such risk is regarded measurable in this method. Human mistake caused by a lack of performance, exhaustion, stress, or poor training is the primary cause of workplace accidents. According to this strategy, personal safety training and education to enhance information and awareness are the best strategies to reduce workplace risk (34). The suggested concept of health capital urges policymakers to recognize the complexities of various health-related resources and assets that individuals hold and deploy to create individual health. Health policies can thus focus on increasing the convergence of healthcare norms and human dispositions, so enhancing the relationship between “public objectives for the good health and good order of the social body with the desire of individuals for personal health and well-being” (33, 35). Based on the qualitative data and our research framework, we developed the following themes for the data analysis and interpretation:

#### 3.2.1. Noise exposure and associated health impacts

The primary reasons of noise on the boats were discussed with fish harvesters. Most interviewees concurred that the primary source of noise is the vessel engine. Fish harvesters stated the following,

*“They are supposed to be the engine and the hydraulics” (FH-2);*

*“The engine that you have in the boat …” (FH-4);*

*“Primary noise would be the main engine, generator …” (FH-6);*

*“The most noise is in the engine room …” (FH-8).*

The harvesters noted that in addition to the engine, hydraulics, winches, haulers, generators, and ropes were the other prominent noise sources. Ten out of twelve participants said their workplace was loud. Harvesters raised their worries about noise on fishing vessels. Fish harvesters outlined the key noise sources,

*“The winch can be noisy when we’re hauling pots because a rope comes up around the rope makes a noise” (FH-1);*

*“I’m running a nine-horsepower system, which requires a nine-horsepower gas utility system, and sometimes that could be very annoying, very loud …” (FH-5);*

*“The diesel engine is on, and the diesel generators are on, so it’s fairly noisy when all of the deck turns on, and all of the hydraulics are on, so it’s noisy when we’re working” (FH-6);*

*“In a small boat, in the 22-footer, I mean you got the outboard motor going on, and you got your hauler motor on, which is fairly loud in just a small area …” (FH-9);*

*“It is always noisy because the generator and motors are on” (FH-10).*

The majority of harvesters (9 out of 12) reported that they experienced no hearing issues. Three harvesters noted hearing loss but had no clue what was causing it. Four of the individuals admitted to experiencing tinnitus. Two participants mentioned that, although they do not have a hearing problem, but are aware of other fishers who do. One fish harvester claimed that exposure to loud noises is the primary cause of tinnitus. They added, “Not very often. When you get a ringing noise in your ears, you usually get it when the noise is too loud” (FH-8). One harvester voiced their views and described how hearing loss among fish harvesters is socially stigmatized. They explained,

*“… if you ask somebody (about the hearing problem), you will get a different response. You’ll say, ‘oh no, my hearing is fine.’ Yes, nobody likes to admit it because there is a stigma around hearing loss. People who can’t hear properly, other people think, oh, people associate hearing loss with intelligence. If somebody can’t hear properly. Well, they’re not very intelligent or something like that, but that’s a social thing” (FH-1).*

Fish harvesters share different health problems while working in a noisy environment. The primary health issues include sleep disturbances, safety risks due to communication difficulties, reduced physical performance, impaired decision-making ability, and changes in voice volume. In addition, some fish harvesters also reported annoyance, irritation, stress, fatigue, headaches and emotional challenges from working in noisy environments.

Some participants indicated that regular exposure to noise at their workplace causes issues. One participant discussed how noise impairs their ability to make decisions by stating the following,

*“Sometimes, the noise bothers me to the point where I do make some rush decisions, but overall, on a scale of 1 to 10, I would have rated 2, maybe 3. Noise doesn’t really influence my decisions to hold on” (FH-5).*

One fish harvester admitted facing emotional challenges due to working in a noisy environment and said, “Yes, sometimes it’s emotional and just 101 wanting to say, like, you know, wanting to give up and just, like, wanting to retire and stuff” (FH7).

Some participants mentioned that being around noise made it harder to communicate and that they had to speak louder to be heard. Fishers said that they do not use hearing protection since it is difficult to communicate, which may result in an accident.

#### 3.2.2. Adaptation in a noisy environment

Most participants agree that their workplace is noisy, but they are habitual to the environment. The thoughts of some of our research participants on workplace noise and associated behavior are as follows: “The diesel engine on, diesel generators on, so fairly noisy on all the deck, turn on all the hydraulics, so it’s noisy when you are working” (FH-6);

*“In the small boat, in the 22-footer, I mean you got the outboard motor going on, and you got your hauler motor on, which is fairly loud in just a small area” (FH-9);*

*“The loudest noise exposure to when we were moving from one string to the next or when we’re coming from the harbor out to the crab fishing grounds, and that’s when the engine is running full, full RPM (rotations per minute), and it’s that’s it loud, and so, if you are on the deck, it can be noisy” (FH-1);*

*“It’s always noisy, because the generator and motors are on” (FH-10).*

One fisher shared their ideas, highlighting the adaptability of fish harvesters in noisy workplace.

*“… I have been fishing all my life, like fishing for 30 or 40 or 50 years. They (fish harvesters) used to do what they do and will do for the next five or ten years. I don’t think it is going to make a great deal of difference anyway, so as you’re getting older and if you have hearing loss if you have been here for 40 years, you’re going to be inclined like this” (FH-2).*

The perspectives of fish harvesters indicate that they have been accustomed to loudness and have adjusted to it, which is a sign of fatalistic behavior.

#### 3.2.3. Limited noise preventive measures

Most fish harvesters do not use hearing protectors when operating on fishing boats. When asked how fish harvesters cope with workplace noise, they responded differently. Three fish harvesters reported that they use earplugs when going inside the engine compartment. One participant noted that they usually move away from the noise’s source. Two harvesters indicated that they would turn off the hauler power and move away from the noisiest place whenever possible.

#### 3.2.4. Safety and health: A conflicting value

Most fish harvesters stated that safety is their top priority and that wearing hearing protection may risk their safety. Use of hearing protectors increases the risk of falling overboard, miscommunication between workers, and other health safety issues. According to a fisher,

*“The challenges on our fishing vessels are that when you wear a hearing device to block the noise, you are also blocking other people who are working around you from hearing what they are saying, and if somebody falls over the boat, and they are trying to sing out to the captain, and he got a hearing device and can’t hear, and that could be a major problem.” (FH-3).*

One other participant explained, “The obstacles, like I have been saying is, having that protection to protect yourself, but also being able to hear somebody and when something has happened in … “(FH-9).

#### 3.2.5. Lack of safety training

Ten out of twelve fish harvesters noted that noise was not considered during their training and management programs. Two harvesters highlighted that some training sessions included occupation exposure of noise and preventive measures. According to a harvester,

*“No, no, not that I know of, the training courses, I took part in, nothing really covers hearing or noise protection and anything like that” (FH-9).*

One fisher said, “I do not do lots of courses. I do not recall the actual anything noise safety course” (FH-12).

One harvester highlighted the safety training and said, “I think there is something for Basic safety training that talks about but no specific for noise” (FH-2).

One participant said they took seminars in survival skills from the Canadian Coast Guard, but none of the sessions mentioned noise exposure and related health impacts.

#### 3.2.6. Barriers and challenges in noise prevention

Various factors hamper the reduction of noise exposure and prevention of hearing damage. The harvesters emphasized the value of better safety regulations, innovative ship structural design, training, and education. Fishers also underlined the need for fish vessel owners and operators to provide enough personnel protection equipment. One fisher stressed the necessity of raising public knowledge about noise exposure and its effects on health. According to the fisherman,

*“I believe a significant portion of it is simply education, becoming educated and aware of the problem and how to prevent it,” (FH-1).*

Some fishers recognized the significance of recent advancements in ship design to reduce noise exposure at fishing vessels.

One fish harvester mentioned, “For winches, they used to be with gas-operated winches and generators. Now, we are coming out with electric, what we notice, cut down the noise a big time” (FH-2). Another harvester recommended making the present diesel engine and generator quieter.

#### 3.2.7. Gaps in OHS regulations

Fish harvesters indicated that provincial organizations need to put more effort in training and education of fishers. One harvester mentioned, “I mean, I never heard of WorkplaceNL putting many efforts into the fishery, and I never heard of anything or any program or meetings there are going on and talking about the fisheries” (FH-9).

One fisher suggested enforcement of OHS regulation mandating protective gear when necessary. One participant stated, “…try to introduce new mandatory hearing protection, maybe from the federal-provincial government or WorkplaceNL even” (FH-5).

Harvesters proposed that ship operators and workers should be required to undergo training and seminars on hearing conservation. One fisher recommended that they get their hearing checked regularly (FH-1). Additionally, fish harvesters lacked enough PPEs on board (FH-5, FH-7, FH-11). One harvester said, “I got the earmuffs for myself, but I do not have them for other crew members, and I do take them with me “(FH-7).

Most fishers were unaware of any fishing groups in NL offering assistance with noise-related problems. Some fish harvesters had no idea that any compensation covered them for health issues caused by noise.

## 4. Discussion

Noise is a significant health risk for fish harvesters. In the current study, we observed how occupational noise exposure affects fish harvesters’ health and well-being and how altered behavior and adaptation in a hazardous environment can lead to long-term disability, such as hearing impairment. The study also shows how improper policy and regulation enforcement can have a negative impact on health and force workers to adapt to a hazardous environment. According to the study findings, fish harvesters operate in a noisy workplace and occasionally use hearing protection. This is also confirmed by a study on noise exposures on small fishing vessels by (36) where the authors performed occupational noise exposure measurements. The restricted usage of hearing protection was due to safety concerns such as falling overboard, miscommunication, and accidents. The research reveals that fishers adapted to their noisy environment and learned to endure the noise. The study also found that NL fish harvesters lacked knowledge and understanding of occupational noise exposure and the associated health hazards.

The concept of relating an individual’s health to capital dates to political economy debates, beginning with Mushkin’s (37) view of health as an investment and Becker’s (38) view of health as a component of human capital. Grossman (39) expands on these perceptions by coining the term “health capital” as a component of a demand model for the product “good health.” This view identifies health as a “durable capital stock that produces an output of healthy time” that drops with age but can be invested *via* medical treatments (33). The health capital approach helps in understanding the value of health as an investment and guides us in avoiding the various individual risks we encounter.

Fishers operate in a confined, mobile, and noisy environment for several days, which affects their health and well-being. Noise from many sources, such as engines, haulers, hydraulic systems, ropes, generators, and the environment, results in various health risks. There are two types of noise transmission on vessels, structure-borne and airborne noise transmission (40). Each fishery has its own set of machinery since different fishing techniques and gear are used to catch different species (40). According to the literature, the greatest noise levels recorded in engine rooms ranged from 56 to 114 dB (5, 10, 11, 14, 34, 41). A large retrospective research was recently carried out among French commercial seafarers to emphasize hearing impairment. The study results are consistent with our findings, which indicate that working in an engine room is a significant risk factor for hearing impairment (42).

Training sessions can improve understanding and awareness of the risk posed by noise. None of the participants could link their general health issues to noise exposure. Many fish harvesters claimed to have attended general safety training/seminars, but deny receiving any particular training on noise exposure and the related health risks. According to WorkplaceNL, all employees who are overly subjected to noise must be educated and trained to ensure they understand the program, the health risks associated with noise, the noise levels in the workplace, and the controls that are in place. Workers must be educated and trained on the selection, fitting, usage, care, and maintenance of hearing aids if it is used. Such instruction and training are typically delivered concurrently with yearly hearing tests. It is also critical for the OHS committee/Workplace Health & Safety representative to attend this instruction (43).

Tinnitus and NIHL are the most common hearing health issues among fish harvesters (34, 44–47). One of the most common neurological symptoms, tinnitus, has been recognized as an early predictor of NIHL and has a prevalence ranging from 19 to 67% (34). Long-term exposure to dangerous noise levels is a known risk factor for NIHL (6). Literature suggests that NIHL is common among fish harvesters and is associated with job duration (10, 44, 48). The prevalence of NIHL was reported 6–80% among fish harvesters globally (34). It is estimated that an exposure to 85 dB(A) noise for 24 h will equal an exposure to 90 dB for 8 h (A). The high and continuous noise exposure leads to hearing loss, sleep problems, blood pressure changes, and the likelihood of accidents (49). According to a Swedish study, many fishers who had their hearing checked, especially those who were still quite young, had impaired hearing. This hearing impairment is considered to be caused mainly by workplace noise exposure (50).

Limited research has been undertaken to describe a direct association between occupational noise exposure and its impact on fish harvesters’ overall health. Sholihah and Satria (51) conducted a study to highlight non-auditory health impacts induced by noise, such as physiological and psychological issues. Physiological disturbances include elevated blood pressure, rapid heartbeat, higher basal metabolism, vasoconstriction of blood vessels, reduced intestinal bowel movement, and elevated muscular tension. Psychological illnesses can add to stress if the sound is unpleasant and upsetting, causing negative sensations, and draining. It can impair focus, emotional issues, sleep disturbances, and communication problems, which negatively impact worker safety (51). Arumugam et al. (52) identified noise exposure as a stressor and noticed common symptoms such as headache (38.09%) and sleep disruptions (7.9%) among research participants. A study conducted by Zeigelboim et al. (53) used a questionnaire survey and clinical examination to explore the health conditions of fish harvesters in Brazil. The authors used electronystagmography to conduct the vestibular examination and assessed self-reported otorhinolaryngological signs and symptoms. Around half of the fishers shared the symptoms of dizziness and headache. Clinical examination revealed other non-auditory health issues, including fatigue, anxiety, depression, and sleep disturbances.

Fish harvesters believe that regulations limiting noise exposure and mandating the use of hearing protection should be implemented for large boats, but research shows that small boats generate more noise than is recommended. According to a recent study on fish harvesters in Newfoundland and Labrador, loud noise levels that can be detrimental are frequently present around small fishing vessels (40). The majority of fishing boats in Atlantic Canada are small vessels, but international fishing OHS requirements primarily pertain to vessels 24 m and above and more than 150 GT. Only the STCW-F Convention, which governs the education and licensing requirements for fishing masters and engineers, has been adopted internationally and ratified by Canada. In an attempt to improve the implementation of health and safety regulations on fishing boats and to encourage safety training among fishing employees, WorkSafeNB has recently worked to modify the Occupational Health and Safety Act. However, raising safety standards will raise running expenses for small fishing boats operated by families, which could impede the success of this regional fishing health and safety program (54). Burella et al. (39) used a job-based method to assess the 8-h equivalent L_EX,8h_ noise level among NL fish harvesters. The measured values were matched to the province’s appropriate NIHL risk criteria. The authors identified that fish harvesters were frequently exposed to hazardous noise levels; fishing operations involving the utilization of hydraulic deck machinery, such as winches and fish pumping systems, as well as the involvement of repeated gear impacts and the usage of outboard engines, were also responsible for harmful noise levels, and skippers were not fully aware of the excessive noise hazards on board their vessels. The authors developed recommendations for technical solutions and types of hearing protection devices and applications to minimize noise exposures based on these findings. The authors suggested the regular use of hearing aids, the need for a change in the acoustic design of vessels, and the implementation of awareness programs for fish harvesters (39). In the present research, most participants identified the engine as a source of noise exposure. However, in the study conducted by Burella et al. (39), fish harvesters also described other sources of noise exposure, such as winches and fish pumping systems.

The current study focuses on the behavior of fish harvesters in tolerating noise and adapting to the loud environment. Information, education, and training on occupational exposure to noise and associated health impacts are much needed to make fish harvesters aware of the risk of noise on their health and well-being. Fish harvesters also noted the inadequate supply of PPEs aboard. The employer must have the appropriate hearing protection equipment for fish harvesters while working in a loud environment. Fish harvesters said they do not wear hearing aids because of other safety issues, including poor communication, falling overboard, and being involved in accidents. Fish harvesters might not be the best candidates for conventional hearing aids. In order to interact with other team members efficiently, they could block out too much sound. There are ear muffs and ear plugs that reduce noise while allowing for the ability to hear other persons and equipment (55). WorkSafeBC recommends that fish harvesters use these specialized hearing-protecting devices while sleeping to protect themselves from loud noises. According to WorkplaceNL, employees need instruction and training on the proper fit, use, and maintain a hearing device. The completion of such education and training frequently occurs around a worker’s yearly hearing test (56). These might be interim methods to reduce noise exposure among fish harvesters. Long-term solutions should focus on improving the acoustic design of fishing vessels, improving insulation, and providing safe noise levels inside cabins (57). According to International Labour Organization, the responsible authority must take action to reduce excessive noise and disturbance in sleeping areas and, to the greatest extent possible, in line with the applicable international standards. The responsible authority must implement noise and vibration standards for accommodation areas for boats 24 meters and longer so that fishermen are adequately protected from the effects of noise and vibration (58). To some extent, all noise issues can be solved, and remedies typically fall into the following categories: Noise reduction at the cause—equipment and machinery selection, attention to precision in machinery installation; Noise insulation at the source, e.g., barriers, separation; Noise shielding in loud sections, e.g., engine room; Workplace or accommodation insulation and Provision of earplugs (41).

*Maritime Occupational Health and Safety regulations (MOHS)* (59) cover health and safety issues, including onboard noise control and prevention measures. According to Maritime OHS standards (59), a person must not be subjected to a continuous noise level in crew accommodation that exceeds 75 dB(A). Employers must also appoint an expert to assess the noise exposure level and notify the worksite committee or the health and safety officer of the investigation if it is not possible for the employer to keep an employee’s exposure to a noise at or below the recommended level. These regulations apply to vessels registered in Canada and are only applicable to seafarers working in Canada. Similarly, the IMO’s code on noise level aboard ship criteria recommends noise levels [dB(A)] in the accommodation area range from 60 to 75 dB (A) (17). These are rules applicable on merchant vessels, while limited provisions were provided for people working on fishing vessels. Noise prevention guidelines for fishing vessels should be designed dependent on the MOHS standards.

According to a safety report published by the TSB (60), noise is one of the critical environmental factors responsible for fatigue among fish harvesters. Traditional methods of controlling fatigue in fishery include controlled work/rest schedules. Work/rest times are addressed under the *Marine Personnel Regulations*; however, this applies to fishing vessels of more than 100 GT. Fishers do not obtain adequate sleep if vessel movement and noise disrupt their sleep (60). Noise exposure recorded in a provincial government-sponsored research found high noise levels on shrimp vessels. Noise exposure aboard fishing vessels may be reduced through short-term measures, long-term strategies, and fish harvester’s education and training (61). Short-term solutions include vessel adaption and the use of PPEs. In contrast, long-term solutions involve vessel renovation or new vessel design and the development of strategies to bring noise levels down to tolerable levels (61).

The research contributes to the existing body of information on noise exposure and related health risks in fish harvesters. The current study findings add information to fish harvesters, fishing organizations, safety instructors, and regulatory agencies. However, due to data collecting time constraints and COVID-19 restrictions, we could only complete 12 interviews with limited female fish harvesters’ representation. Additionally, because the research was only performed in a limited geographic area (Newfoundland and Labrador), it is challenging to extrapolate the study results to other regions of the world. To further assess onboard noise risk and related health implications among fish harvesters in the future, in-person interviews and focus groups, might be undertaken with a larger sample encompassing a wider geographic region.

## 5. Conclusion

The current research findings indicate that onboard noise exposure is a significant health issue and must be addressed by the relevant authorities promptly. Fish harvesters know that their workplace is noisy but still have to work with a limited supply of hearing protective devices onboard. The research also identifies gaps in how employers have implemented provincial *OHS regulations*. Owners and operators should take responsibility to ensure that fish harvesters have access to enough PPE and abide by all other regulations to manage noise exposure and prevent hearing damage. Newer technology can be adopted during the ship design to minimize noise exposure. All the stakeholders, including fishing organizations, the provincial government, and the federal government, should provide additional training and education so that fish harvesters can better understand the noise risk and related health impacts.

## Data availability statement

The raw data supporting the conclusions of this article will be made available by the authors, without undue reservation.

## Ethics statement

The studies involving human participants were reviewed and approved by Interdisciplinary Committee on Ethics in Human Research (ICEHR), Memorial University of Newfoundland. The patients/participants provided their written informed consent to participate in this study.

## Author contributions

OY, DS, AS, and LM: conceptualization. OY, DS, and AS: methodology. OY: validation, data curation, writing-original draft preparation, and visualization. OY and DS: formal analysis. DS, AS, and LM: writing-review and editing and supervision. DS and LM: project administration and funding acquisition. All authors contributed to the article and approved the submitted version.

## Funding

This research received funding from Future Ocean and Coastal Infrastructures (FOCI), an Ocean Frontier Institute project. The research funding was provided by the Ocean Frontier Institute, through an award from the Canada First Research Excellence Fund. Mitacs, Canada through Mitacs research training award, Dean’s special award, Faculty of Medicine at Memorial University of Newfoundland, and Graduate Research Funding at Memorial University of Newfoundland.

## Conflict of interest

The authors declare that the research was conducted in the absence of any commercial or financial relationships that could be construed as a potential conflict of interest.

## Publisher’s note

All claims expressed in this article are solely those of the authors and do not necessarily represent those of their affiliated organizations, or those of the publisher, the editors and the reviewers. Any product that may be evaluated in this article, or claim that may be made by its manufacturer, is not guaranteed or endorsed by the publisher.
